# Development and validation of risk-adjusted quality indicators for the long-term outcome of acute sepsis care in German hospitals based on health claims data

**DOI:** 10.3389/fmed.2022.1069042

**Published:** 2023-01-09

**Authors:** Lisa Wedekind, Carolin Fleischmann-Struzek, Norman Rose, Melissa Spoden, Christian Günster, Peter Schlattmann, André Scherag, Konrad Reinhart, Daniel Schwarzkopf

**Affiliations:** ^1^Institute of Medical Statistics, Computer and Data Sciences, Jena University Hospital, Jena, Germany; ^2^Institute for Infectious Diseases and Infection Control, Jena University Hospital, Jena, Germany; ^3^Integrated Research and Treatment Center for Sepsis Control and Care, Jena University Hospital, Jena, Germany; ^4^Department of Anesthesiology and Intensive Care Medicine, Jena University Hospital, Jena, Germany; ^5^Federal Association of the Local Health Care Funds, Research Institute of the Local Health Care Funds (WIdO), Berlin, Germany; ^6^Department of Anaesthesiology and Operative Intensive Care Medicine (CCM, CVK), Charité Universitätsmedizin Berlin, Corporate Member of Freie Universität Berlin, Humboldt-Universität zu Berlin, Berlin, Germany; ^7^Campus Virchow-Klinikum, Berlin Institute of Health, Berlin, Germany

**Keywords:** sepsis, mortality, risk-adjustment, administrative claims, diagnosis related groups, health care quality assessment

## Abstract

**Background:**

Methods for assessing long-term outcome quality of acute care for sepsis are lacking. We investigated a method for measuring long-term outcome quality based on health claims data in Germany.

**Materials and methods:**

Analyses were based on data of the largest German health insurer, covering 32% of the population. Cases (aged 15 years and older) with ICD-10-codes for severe sepsis or septic shock according to sepsis-1-definitions hospitalized in 2014 were included. Short-term outcome was assessed by 90-day mortality; long-term outcome was assessed by a composite endpoint defined by 1-year mortality or increased dependency on chronic care. Risk factors were identified by logistic regressions with backward selection. Hierarchical generalized linear models were used to correct for clustering of cases in hospitals. Predictive validity of the models was assessed by internal validation using bootstrap-sampling. Risk-standardized mortality rates (RSMR) were calculated with and without reliability adjustment and their univariate and bivariate distributions were described.

**Results:**

Among 35,552 included patients, 53.2% died within 90 days after admission; 39.8% of 90-day survivors died within the first year or had an increased dependency on chronic care. Both risk-models showed a sufficient predictive validity regarding discrimination [*AUC* = 0.748 (95% CI: 0.742; 0.752) for 90-day mortality; *AUC* = 0.675 (95% CI: 0.665; 0.685) for the 1-year composite outcome, respectively], calibration (Brier Score of 0.203 and 0.220; calibration slope of 1.094 and 0.978), and explained variance (*R*^2^ = 0.242 and *R*^2^ = 0.111). Because of a small case-volume per hospital, applying reliability adjustment to the RSMR led to a great decrease in variability across hospitals [from median (1st quartile, 3rd quartile) 54.2% (44.3%, 65.5%) to 53.2% (50.7%, 55.9%) for 90-day mortality; from 39.2% (27.8%, 51.1%) to 39.9% (39.5%, 40.4%) for the 1-year composite endpoint]. There was no substantial correlation between the two endpoints at hospital level (observed rates: ρ = 0, *p* = 0.99; RSMR: ρ = 0.017, *p* = 0.56; reliability-adjusted RSMR: ρ = 0.067; *p* = 0.026).

**Conclusion:**

Quality assurance and epidemiological surveillance of sepsis care should include indicators of long-term mortality and morbidity. Claims-based risk-adjustment models for quality indicators of acute sepsis care showed satisfactory predictive validity. To increase reliability of measurement, data sources should cover the full population and hospitals need to improve ICD-10-coding of sepsis.

## 1. Introduction

Sepsis is the final pathway to death from infectious diseases (1) and affects an estimated 49 million patients per year worldwide, of whom 11 million die (2). It is considered as one of the leading causes of preventable deaths in hospitals (3). One-sixth of sepsis survivors experience severe persistent physical disability or cognitive impairment, and one-third die during the following year after the acute disease (4). Acknowledging deficits of care, the World Health Assembly adopted the sepsis resolution WHA70.7 in May 2017, which urges WHO member states to improve prevention, diagnosis and management of sepsis (5).

Measuring and comparing performance of health care providers are a central part of quality improvement (6). For this purpose, administrative health data can be used for performance measurement with the advantage of covering all ICD-coded cases with data readily available, at minimal time and costs (7). Performance measures need to account for differences in the mix of important patient attributes across hospitals by adequate risk-adjustment models (8, 9). Several risk-adjusted quality indicators on sepsis care based on administrative health data have been presented in the literature (10–13). Such indicators have been used to assess and compare hospital performance as well as to evaluate effects of voluntary and mandated quality improvement programs (14, 15). Existing administrative data-based indicators on the quality of sepsis care share two shortcomings. First, they only used in-hospital or 30-day post-discharge mortality as outcomes, although short-term case fatality is increasingly regarded to be inadequate as sole metric for the outcome of sepsis patients (4, 16). Improved quality of care should ideally reduce short-term mortality, but also long-term mortality and morbidity resulting in a higher proportion of patients with full recovery. Second, risk-adjustment models were based on hospital discharge data solely. Therefore, pre-existing conditions were defined only based on ICD-coding during the hospital stay, which may result in bias based on incomplete coding as well as a failure to distinguish conditions present-on-admission from complications (7, 17, 18).

To overcome these shortcomings, we developed risk-adjusted quality indicators based on longitudinal health claims data incorporating long-term outcomes of sepsis care as well as pre-existing conditions coded before hospital admission.

## 2. Materials and methods

### 2.1. Data source

This is a secondary analysis of health care claims data provided for the SEPFROK study (19). This cohort study was based on nationwide anonymous administrative health claims data of the largest German health insurance, the “Allgemeine Ortskrankenkasse” (AOK), which covers approximately 32% of the German population. Data were provided by the Research Institute of the Local Health Care Funds (WIdO). Health insurance is mandatory in Germany; residents can select any insurer and enroll without restriction. Within the AOK data, hospitals were identified by a unique institutional identifier (IK: “Institutionskennzeichen”). More than one hospital site of the same institution might use the same IK, but typically, these sites are organizationally linked and mutually dependent. Based on the AOK health care claims data, the WIdO already provides the quality assurance using health claims data “Qualitätssicherung mit Routinedaten” (QSR, quality assurance using routine data) (20). Indicators are reported to participating hospitals and are part of a web-based information portal to support patients in selecting a hospital. Sepsis is not yet part of the set of quality indicators.

### 2.2. Study population

The SEPFROK study included patients aged ≥ 15 years with an inpatient hospitalization (discharged January 1, 2013, to December 31, 2014) with an explicit *International Statistical Classification of Diseases and Related Health Problems, Tenth Revision, German Modification* (ICD-10-GM) code for sepsis as primary or secondary discharge diagnoses (Supplementary material 1–Definition of variables). The first hospitalization with sepsis within this period was defined as the index hospitalization and included in the analyses. Patients with a diagnosis of sepsis in the 2 years preceding the index hospitalization were excluded. Since SEPFROK included a 5-year-look-back period and a 3-year-follow-up, patients who were not continuously insured from January 1, 2009, through their respective 3-year follow-up period after the index hospitalization (or until death) were excluded (Supplementary Figure 1).

For this secondary analysis, we included patients with index hospitalization with severe sepsis or septic shock defined by ICD-Codes R65.1 and R57.2 in 2014.

### 2.3. Outcomes

We included short- and long-term endpoints. 90-day mortality after hospital discharge was chosen as short-term endpoint. As long-term outcome, we defined a composite (binary) outcome of 1-year mortality and increase in the dependency on chronic care during the year after hospital discharge from index hospitalization to address the competing risk they represent (21). The increase in dependency on nursing care was defined by an increase in nursing care level or a new transition to a long-term nursing home, which both are recorded with high reliability in claims data and thus can serve as objective measure of a relevant increase of morbidity and decrease of functioning. In Germany, nursing care levels are defined on graded care needs and entitle patients to long-term care insurance benefits. Care can be provided by informal or formal caregivers or in nursing homes (see Supplementary material 1: Definition of variables for details). For the analysis of the composite endpoint only 90-day survivors were included.

### 2.4. Model derivation

#### 2.4.1. Risk factors

Based on clinical reasoning and existing research, candidate variables were chosen among patient demographics, pre-existing comorbidities, pre-existing conditions and treatments, clinical characteristics of the infection, hospital admission type and specific treatments during the index hospitalization (10–12, 22–24). Detailed definitions of risk factors are given in detail in the Supplementary material 1.

##### 2.4.1.1. Patient demographics

Patient demographics included gender and age. To allow for non-linear effects of age, quadratic, and cubic polynomials were included. Age was transformed by mean-centering and standardization to decades [age_*t*_ = (age–70)/10].

##### 2.4.1.2. Comorbidities

Comorbidities were assessed in a period of 12 months prior to hospitalization and were defined by the categories of the Charlson Comorbidity Index (CCI) and the Elixhauser Comorbidity Index (ECI) (25, 26) based on a German adaptation of a previously developed ICD-10 coding algorithm (27, 28). If a CCI and an ECI category assessed the same comorbidity, the ECI category was included. An additional indicator variable for presence of leukemia was also included.

##### 2.4.1.3. Pre-existing conditions and treatments

Pre-existing conditions and treatments included the prior dependency on immobility, nursing care, mechanical ventilation, renal replacement therapy, palliative care, which were defined by procedures and general medical measures [OPS: Operationen und Prozedurenschlüssel (German Procedure Classification)] and ICD-10-GM codes, were assessed in a period of 12 months prior to the index hospitalization. The cumulative length of previous hospital stays during the 1-year period before the index hospitalization was categorized as follows: “0 day,” “1 day,” “> 1 day and < 6 days,” “≥ 6 days and ≤ 10 days”, and “> 10 days.”

##### 2.4.1.4. Clinical characteristics of the infection

Clinical characteristics of the infection included “focus of infection” defined by presence of specific ICD-10-GM codes, and presence of an explicit sepsis code as a primary diagnosis. “Focus of infection”-codes were derived from the literature (29–31) and clinical knowledge. A primary diagnosis of sepsis was defined if an explicit sepsis code (A40.–A41., R57.2) was present as primary diagnosis. Finally, infection by multi-resistant pathogens was defined by presence of an ICD-10-GM and OPS-code for the presence and treatment of multi-drug resistant pathogens during the index stay.

##### 2.4.1.5. Hospital admission type

Hospital admission type was categorized as “emergency admission,” “referral by physician” or “transfer from another hospital.”

##### 2.4.1.6. Specific treatments during the index hospitalization

Specific treatments during the index hospitalization not related to sepsis care but associated with increased risk of death were also included and defined by OPS-codes (chemotherapy, stroke treatment).

#### 2.4.2. Model development

Two risk-models were developed–one for each specified endpoint. Risk factors were first selected from the set of candidate variables by a logistic regression model with backward elimination for each endpoint. Because of the large sample size, the criterion to exclude variables from the model was set *p* > 0.01. Since patients with septic shock or sepsis as primary diagnosis are a distinctive subgroup with a special importance for quality measurement, we aimed to make the model suitable also for comparing the endpoints within the subgroups of cases with or without septic shock and with or without sepsis as primary diagnosis. Risk factors might have different effects within these respective subgroups, which can be modeled by statistical interaction effects. Therefore, interaction effects of the selected predictors with the presence of a diagnosis of septic shock or sepsis as primary diagnosis were also included and backward-selected in a second modeling step. Since observed outcomes are expected to be correlated within-hospitals, these models were then refitted by hierarchical generalized linear models (HGLMs) with binomial errors, a logit link and a random intercept for the hospitals (9, 32).

#### 2.4.3. Model validation

We did not conduct a validation in external cohorts, since this model is not intended for use in external cohorts. If such a model is intended to be used in a quality assurance program, like QSR, a recalculation on a yearly base would be necessary. Therefore, we conducted an internal validation with correction for over-fitting using two bootstrap approaches. First, following advice by Harrell et al. (33), two hundred bootstrap replications were done by sampling over the hospitals. In each bootstrap step, the variable selection and the re-fitting by HGLM was repeated within the bootstrap sample. The following validation measures were calculated: the area under the curve (AUC) as a measure of discrimination, the squared Pearson correlation (*R*^2^) as measure of explained variation (34, 35), and the Brier Score and the calibration slope as measures of calibration. These validation measures were calculated in the bootstrap sample on the one hand and in the original sample on the other hand. The difference of these two values is the optimism. The corrected performance is the difference of the validation measure in the original sample and the averaged optimism, respectively. The second approach was similar, but estimated validation measures by prediction on the out-of-bag samples in each bootstrap step and then taking their mean (36). To visualize calibration, the distribution of observed mortality across deciles of predicted mortality was plotted.

#### 2.4.4. Calculation of risk-and reliability-adjusted indicators

Risk-adjusted endpoints per hospital were calculated as risk-standardized mortality rates (RSMR). Note that the expression RSMR will also be used when referring to the composite endpoint. Two methods were used to calculate RSMR. The first method was based on the standard logistic regression approach, in which the RSMR results from the ratio of observed mortality to mortality predicted from the logistic regression model, multiplied with the unadjusted rate in the full sample (37, 38). Low number of cases per hospital cause unreliability in the estimation of the RSMR, which results in higher rates of randomly extreme values among hospitals with small case numbers. Reliability adjustment by shrinkage estimators was repeatedly proposed to achieve more stable estimates (39–41). To implement this, we applied the methodology used for the quality indicators of the Centers for Medicare and Medicaid Service’s as a second method (35, 38, 42). Here, reliability-adjusted RSMR are obtained as the ratio of predicted to expected mortality obtained from the HGLM, multiplied by the unadjusted rate of the full sample (38). Confidence limits (95% CI) were calculated by a large sample approximation for the RSMR (37, 38), and by a bootstrap approach, as described by Normand et al. (42), for the reliability-adjusted RSMR. The distribution of the observed rates, RSMR and reliability-adjusted RSMR was analyzed by descriptive statistics and graphics. The bivariate relationships between the 90-day mortality and the composite endpoint of 1-year mortality or increased dependency on nursing care was analyzed by scatterplot and calculation of Pearson’s ρ for unadjusted rates, RSMR and reliability-adjusted RSMR, respectively. Hospitals with a CI not overlapping with the unadjusted rate of the full sample are regarded as showing a deviation from the average. We used cross-tabulation to describe the distribution of hospitals with a significant deviation in RSMR or reliability-adjusted RSMR for both endpoints. The data analysis for this paper was generated using SAS software, version 9.4 (Copyright© 2002–2012 SAS Institute Inc., SAS and all other SAS Institute Inc., product or service names are registered trademarks or trademarks of SAS Institute Inc., Cary, NC, USA) and with R, version 4.1.1 [R Core Team, 2020 (43). R: A language and environment for statistical computing. R Foundation for Statistical Computing, Vienna, Austria. URL https://www.R-project.org/].

## 3. Results

Table 1 provides an overview of the study sample (and Supplementary Figure 1 is the corresponding flowchart). Among 35,522 patients who were hospitalized with sepsis or septic shock in 2014, 18,884 (53.2%) died within the first 90 days after admission. Of 16,638 90-day survivors, 3,632 (21.8%) died within the first year. Increase in the dependency on nursing care affected 4,316 (25.9%) of 90-day survivors. Death or increase in the dependency on nursing care (composite endpoint) occurred in 6,639 (39.8%) of 90-day survivors. The mean age of the total sample at hospital cases was 73.96 ± 12.28 years (90-day survivors: 71.58 ± 13.02 years), 45% were males (90-day survivors: 44.7%). Patients of the total sample were treated in 1,174 hospitals. Since part of hospitals had no 90-day survivors, the 1-year composite endpoint was analyzed for 1,105 hospitals only.

**TABLE 1:** Sample of cases with severe sepsis or septic shock.

Cases	N (%)
Hospitalized cases with severe sepsis or septic shock in 2014	35,522 (100%)
90-day deceased	
n (% of cases with severe sepsis or septic shock)	18,884 (53.16%)
90-day survivors	
n (% of cases with severe sepsis or septic shock)	16,638 (46.84%)
1 year mortality	
n (% of cases which survived 90 days)	3,632 (21.83%)
1 year survivors	
n (% of cases which survived 90 days)	13,006 (78.17%)
increased dependency on chronic care	
n (% of cases which survived 90 days)	4,316 (25.94%)
no increased dependency on chronic care	
n (% of cases which survived 90 days)	12,322 (74.06%)
1 year composite endpoint (1 year mortality OR increased dependency on chronic care)	
n (% of cases which survived 90 days)	6,639 (39.8%)
no 1 year composite endpoint (1 year mortality OR increased dependency on chronic care)	
n (% of cases which survived 90 days)	10,029 (60.2%)

### 3.1. Risk-adjustment models

After backward selection 29 of 58 initial risk factors and 12 of 136 initial interaction effects with septic shock or sepsis as primary diagnosis were identified for the endpoint 90-day mortality. Higher age, emergency admission or transfer from another hospital, and septic shock were associated with increased risk of death. In general, indicators of preexisting morbidity–like pre-sepsis comorbid illness, duration of previous hospital stays, or pre-existing treatments–were associated with an increased risk of death. Exemption were depression, complicated hypertension, obesity, and pre-existing long-term ventilation, which showed protective effects (Table 2). For the 1-year composite endpoint (1-year mortality or increased dependency on nursing care), 27 risk factors and 10 of 112 initial interaction effects with septic shock or sepsis as primary diagnosis were identified. Again, higher age, emergency admission or transfer from another hospital, and septic shock were associated with increased risk. Indicators of pre-sepsis morbidity were all associated with increased risk, with the exemption of pre-existing dependency on chronic care, which showed a protective effect (Table 2).

**TABLE 2:** Coefficients estimates of the risk-adjustment model for 90-days mortality and 1-year composite endpoint of mortality or increased dependency on chronic care.

	90-days mortality			1-year composite endpoint of mortality or increased dependency on chronic care		
**Variable**	**Mean ± SD or%**	***P*-value**	**Odds ratio [95% CI]**	**Mean ± SD or%**	***P*-value**	**Odds ratio [95% CI]**
**Patient demographics**						
Age^a^	73.96 **±** 12.28			71.58 ± 13.02	< 0.001	1.49 [1.44; 1.53]
*Effect in non-primary diagnosis of sepsis*		< 0.001	1.44 [1.39; 1.50]			
*Effect in primary diagnosis of sepsis*		< 0.001	1.33 [1.27; 1.39]			
Age^2^		< 0.001	1.05 [1.04; 1.07]			
Age^3^		< 0.001	1.01 [1.01; 1.02]			
**Comorbidities**						
Charlson: Dementia	20.09%			16.20%	< 0.001	1.31 [1.18; 1.45]
*Effect in non-primary diagnosis of sepsis*		0.886	1.01 [0.92; 1.11]			
*Effect in primary diagnosis of sepsis*		< 0.001	1.26 [1.15; 1.39]			
Charlson: Moderate or severe liver disease	2.44%	< 0.001	1.87 [1.59; 2.20]			
Elix: Alcohol abuse	9.22%	< 0.001	1.29 [1.18; 1.40]	8.87%	< 0.001	1.39 [1.24; 1.57]
Elix: Congestive heart failure	43.03%	< 0.001	1.12 [1.06; 1.19]	38.37%	< 0.001	1.16 [1.08; 1.25]
Elix: Depression	28.26%	0.002	0.92 [0.87; 0.97]			
Elix: Diabetes, uncomplicated				44.32%	< 0.001	1.13 [1.05; 1.21]
Elix: Fluid and electrolyte disorders	34.32%	< 0.001	1.25 [1.18; 1.32]	28.77%		
*Effect in non-septic shock*					< 0.001	1.27 [1.15; 1.39]
*Effect in septic shock*					0.978	1.00 [0.85; 1.18]
Elix: Hypertension, complicated	26.34%	< 0.001	0.88 [0.83; 0.93]			
Elix: Leukemia	1.77%	< 0.001	1.66 [1.38; 1.98]	1.33%	0.001	1.64 [1.23; 2.18]
Elix: Metastatic cancer	7.71%			5.57%		
*Effect in non-septic shock*		< 0.001	2.48 [2.23; 2.76]		< 0.0001	2.12 [1.77; 2.53]
*Effect in septic shock*		< 0.001	1.46 [1.23; 1.74]		0.048	1.36 [1.00; 1.83]
Elix: Obesity	27.31%					
*Effect in non-septic shock*		< 0.001	0.83 [0.78; 0.89]			
*Effect in septic shock*		0.643	0.98 [0.88; 1.08]			
Elix: Other neurological disorders				14.77%		
*Effect in non-primary diagnosis of sepsis*					< 0.001	1.27 [1.10; 1.46]
*Effect in primary diagnosis of sepsis*					0.730	0.98 [0.85; 1.13]
Elix: Paralysis				9.57%	< 0.001	1.21 [1.07; 1.37]
Elix: Peripheral vascular disorders	33.03%			30.09%	0.035	1.08 [1.01; 1.17]
*Effect in non-septic shock*		0.264	1.04 [0.98; 1.10]			
*Effect in septic shock*		< 0.001	1.23 [1.12; 1.36]			
Elix: Pulmonary circulation disorders	10.01%	0.007	1.12 [1.03; 1.22]			
Elix: Solid tumor without metastasis				18.99%	0.004	1.14 [1.04; 1.25]
Elix: Valvular disease	21.16%	< 0.001	1.11 [1.04; 1.18]			
Elix: Weight loss	8.62%	< 0.001	1.44 [1.32; 1.57]	6.36%		
*Effect in non-primary diagnosis of sepsis*					0.140	1.16 [0.95; 1.40]
*Effect in primary diagnosis of sepsis*					< 0.001	1.66 [1.37; 2.02]
**Pre-existing conditions and treatments**						
Pre-existing immobility				19.32%	< 0.001	1.14 [1.04; 1.25]
Pre-existing dependency on chronic care	39.51%			32.39%		
*Effect in non-primary diagnosis of sepsis*		< 0.001	1.29 [1.23; 1.36]		< 0.001	0.52 [0.48; 0.56]
*Effect in primary diagnosis of sepsis*		< 0.001	1.42 [1.35; 1.49]		< 0.001	0.66 [0.62; 0.70]
*Effect in non-primary diagnosis of sepsis/Septic shock*		< 0.001	1.31 [1.22; 1.42]			
*Effect in primary diagnosis of sepsis/septic shock*		< 0.001	1.16 [1.07; 1.27]			
Pre-existing long-term mechanical ventilation	1.64%	0.004	0.76 [0.64; 0.92]	1.72%	< 0.001	1.53 [1.18; 1.97]
Pre-existing renal replacement therapy	4.77%	0.002	1.20 [1.07; 1.34]			
Hospital length of stay (> 10 d) (reference)	0.43%			0.37%		
Hospital length of stay (0 d)	35.69%			40.42%		
*Effect in non-primary diagnosis of sepsis*		0.455	0.84 [0.53; 1.33]		0.265	0.66 [0.32; 1.37]
*Effect in primary diagnosis of sepsis*		0.560	0.84 [0.46; 1.52]		< 0.001	0.22 [0.09; 0.55]
Hospital length of stay (1 d)	24.68%			24.48%		
*Effect in non-primary diagnosis of sepsis*		0.535	0.86 [0.54; 1.37]		0.186	0.61 [0.29; 1.27]
*Effect in primary diagnosis of sepsis*		0.760	0.91 [0.50; 1.65]		< 0.001	0.26 [0.11; 0.65]
Hospital length of stay (> 1 d and < 6 d)	34.87%			31.26%		
*Effect in non-primary diagnosis of sepsis*		0.608	0.89 [0.56; 1.40]		0.196	0.62 [0.30; 1.28]
*Effect in primary diagnosis of sepsis*		0.784	1.09 [0.60; 1.96]		0.013	0.32 [0.13; 0.79]
*Effect in non-primary diagnosis of sepsis*		0.969	1.01 [0.63; 1.63]		0.389	0.72 [0.34; 1.53]
Hospital length of stay (≥ 6 d and ≤ 10 d)	4.34%			3.47%		
*Effect in primary diagnosis of sepsis*		0.606	1.18 [0.64; 2.17]		0.053	0.40 [0.15; 1.01]
**Clinical characteristics of the infection**						
Primary diagnosis of sepsis	39.15%	0.005	0.34 [0.16; 0.73]	46.99%	0.531	1.45 [0.45; 4.64]
Septic shock	29.70%			20.76%		
*Effect in non-primary diagnosis of sepsis*		< 0.001	3.33 [2.92; 3.80]		< 0.001	1.22 [1.09; 1.37]
*Effect in primary diagnosis of sepsis*		< 0.001	4.52 [3.91; 5.22]		< 0.001	1.45 [1.25; 1.67]
Site of infection: Abdominal	21.60%					
*Effect in non-septic shock*		0.363	0.97 [0.90; 1.04]			
*Effect in septic shock*		< 0.001	0.80 [0.72; 0.89]			
Site of infection: Respiratory tract	47.65%			45.16%	< 0.001	1.30 [1.22; 1.39]
*Effect in non-primary diagnosis of sepsis*		< 0.001	1.14 [1.06; 1.22]			
*Effect in primary diagnosis of sepsis*		< 0.001	1.36 [1.25; 1.47]			
*Effect in non-septic shock*		< 0.001	1.14 [1.06; 1.22]			
*Effect in septic shock*		0.038	0.90 [0.82; 0.99]			
Site of infection: Device-related infections	8.90%	< 0.001	0.68 [0.63; 0.74]	10.62%		
*Effect in non-primary diagnosis of sepsis*					0.646	1.03 [0.91; 1.17]
*Effect in primary diagnosis of sepsis*					< 0.001	1.44 [1.18; 1.76]
Site of infection: other or unspecified	55.61%			60.81%	< 0.001	1.22 [1.14; 1.31]
*Effect in non-septic shock*		< 0.001	0.87 [0.82; 0.92]			
*Effect in septic shock*		< 0.001	0.57 [0.52; 0.63]			
Site of infection: Genitourinary system	29.97%	< 0.001	0.62 [0.59; 0.66]			
Site of infection: Wound/soft tissue infection	6.54%	< 0.001	0.79 [0.72; 0.87]	7.87%		
*Effect in non-primary diagnosis of sepsis*					< 0.001	1.32 [1.12; 1.55]
*Effect in primary diagnosis of sepsis*					0.811	1.02 [0.85; 1.24]
Multi-resistant pathogen				6.55%	< 0.001	1.40 [1.22; 1.60]
**Hospital admission type**						
Reason for admission: Emergency (reference)	60.05%			61.00%		
Transfer from another hospital	7.09%	< 0.001	1.22 [1.10; 1.34]	5.88%	0.579	1.04 [0.90; 1.21]
Referral by physician or other	32.86%	0.034	0.94 [0.89; 1.00]	33.12%	0.012	0.91 [0.85; 0.98]
**Specific treatments during the index hospitalization**						
Chemotherapy in index hospitalization				2.53%		
*Effect in non-primary diagnosis of sepsis*					< 0.001	1.64 [1.29; 2.09]
*Effect in primary diagnosis of sepsis*					0.685	0.91 [0.57; 1.45]
Stroke treatment in index hospitalization				1.03%	< 0.001	2.57 [1.84; 3.59]

Coefficients estimated by a hierarchical generalized linear model with a logit link and random intercept to adjust for clustering of cases in hospitals. Cases, which were hospitalized with severe sepsis or septic shock in 2014, were included. Italic text presents conditional effects in subgroups of cases estimated based on significant interaction effects with the indicators for presence of septic shock and presence of a primary diagnosis for sepsis. CI, confidence interval. Not all main effects or interaction effects were selected by the backward selection algorithm in both models, which results in some empty cells. ^a^Age was standardized by (Age-70)/10.

### 3.2. Validation

**FIGURE 1:**
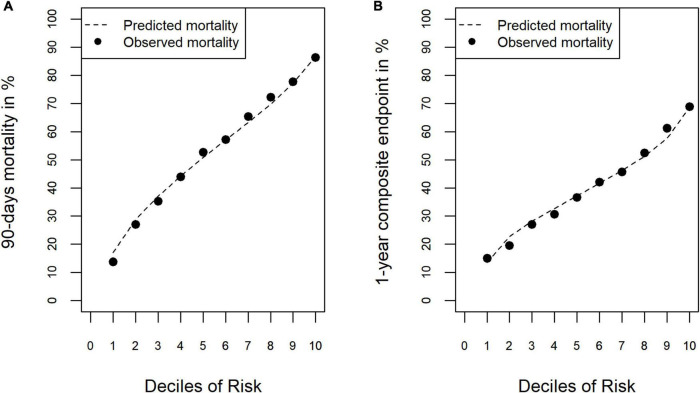
Calibration of the risk-adjustment models. **(A)** 90-day mortality. **(B)** 1-year composite endpoint (1-year mortality or increased dependency on chronic care).

Uncorrected and corrected estimates of the validity measures are given in Supplementary material 2: Internal validation, Supplementary Tables 1, 2. The approach of Harrel et al. resulted in more conservative estimates and yielded a corrected discrimination of *AUC* = 0.748 [95% CI: 0.742; 0.752], an explained variance of *R*^2^ = 0.242, a Brier Score of 0.203 and a calibration slope of 1.094 for the model for 90-day-mortality. The model for the 1-year composite endpoint showed an estimated discrimination of *AUC* = 0.675 [95% CI: 0.665; 0.685], an explained variance of *R*^2^ = 0.111, a Brier Score of 0.220 and a calibration slope of 0.978. Calibration of both models was good (observed rate in lowest risk-decile and highest decile: 0.17–0.87 and 0.13–0.68, respectively, Figures 1A, B).

### 3.3. Distribution of indicators across hospitals

There was a large variation in numbers of cases across hospitals. Across 1,174 hospitals, the number of cases with coded sepsis per hospital for 90-day mortality ranged from 1 to 745, 25th, 50th, and 75th percentile of 7, 17, and 36, respectively. Across 1,105 hospitals, the number of 90-day survivors per hospital for the composite 1-year endpoint ranged from 1 to 334, 25th, 50th, and 75th percentile of 3, 8, and 18, respectively.

**FIGURE 2:**
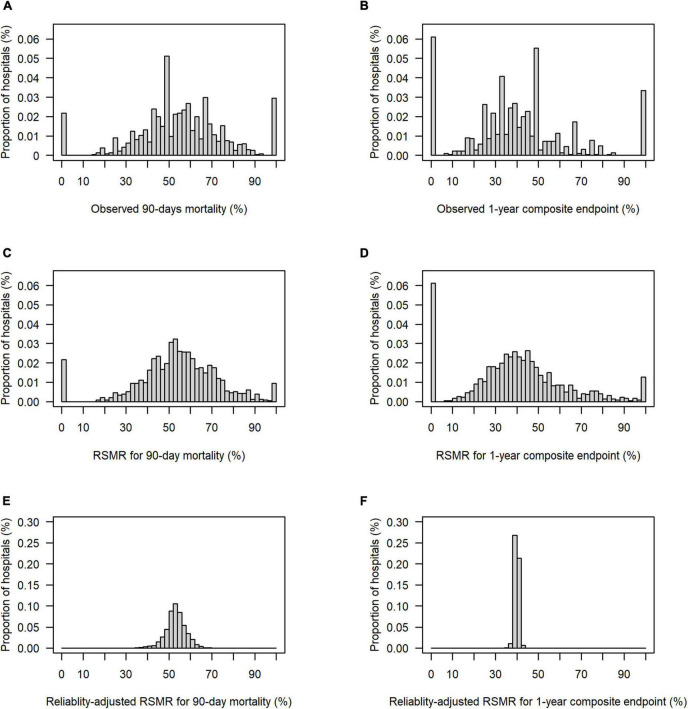
Distribution of observed, risk-standardized and risk-and reliability adjusted endpoints per hospital. **(A)** Observed 90-day mortality, range from 0 to 100%, 25th, 50th, and 75th percentile of 44.0, 54.6, and 66.7%, respectively. **(B)** Observed rate for composite outcome of 1-year-mortality or increased dependency on chronic care, range from 0 to 100%, 25th, 50th, and 75th percentile 26.9, 39.3, and 50.0%, respectively. **(C)** Risk-standardized rate (RSMR) for 90-day mortality without reliability adjustment, range from 0 to 100%, 25th, 50th, and 75th percentiles of 44.3, 54.2, and 65.5%, respectively. **(D)** RSMR for the 1-year composite endpoint without reliability adjustment, range from 0 to 100%, 25th, 50th, and 75th percentiles of 27.8, 39.2, and 51.1%, respectively. **(E)** Reliability-adjusted RSMR for 90-day mortality, range from 35.9 to 68.1%, 25th, 50th, and 75th percentiles of 50.7, 53.2, and 55.9%, respectively. **(F)** Reliability-adjusted RSMR for 1-year composite outcome, range from 35.8 to 43.3%, 25th, 50th, and 75th percentile of 39.5, 39.9, and 40.4%, respectively.

Figure 2 presents the distribution of the two endpoints–90-day mortality and the 1-year composite endpoint per hospital–with their observed values, their risk-adjusted values (RSMR), and their risk-and reliability adjusted values (reliability-adjusted RSMR). While the variability of the RSMR was comparable to the observed values (panel A vs. C, and panel B vs. D, respectively), the implementation of reliability adjustment let to a strong reduction in variability across hospitals (panels E and F).

Reliability adjustment also had an effect regarding the proportion of hospitals, which showed a deviation of the RSMR from average (Table 3). Without reliability adjustment, 7.6% of hospitals showed a RSMR on 90-day mortality with the lower 95% confidence limit above the average; with reliability adjustment, this was true for only 1.2%. Regarding the 1-year composite endpoint, 3.5% of hospitals showed an RSMR above average without reliability adjustment, while none showed a RSMR above average with reliability adjustment.

**TABLE 3:** Proportion of hospitals showing significant deviation of risk-adjusted outcomes from average.

Indicator	Proportion of hospitals with 95% CI of RSMR below the rate in the population	Proportion of hospitals with 95% CI of RSMR including the rate observed in the population	Proportion of hospitals with 95% CI of RSMR above the rate in the population
**RSMR for 90-day mortality**			
Without reliability adjustment	66 (5.62%)	1019 (86.80%)	89 (7.58%)
With reliability adjustment	28 (2.39%)	1132 (96.42%)	14 (1.19%)
**RSMR for 1-year composite endpoint**			
Without reliability adjustment	20 (1.81%)	1046 (94.66%)	39 (3.53%)
With reliability adjustment	0 (0.0%)	1105 (100.0%)	0 (0.0%)

RSMR, risk-standardized mortality rate; CI, confidence interval.

**FIGURE 3:**
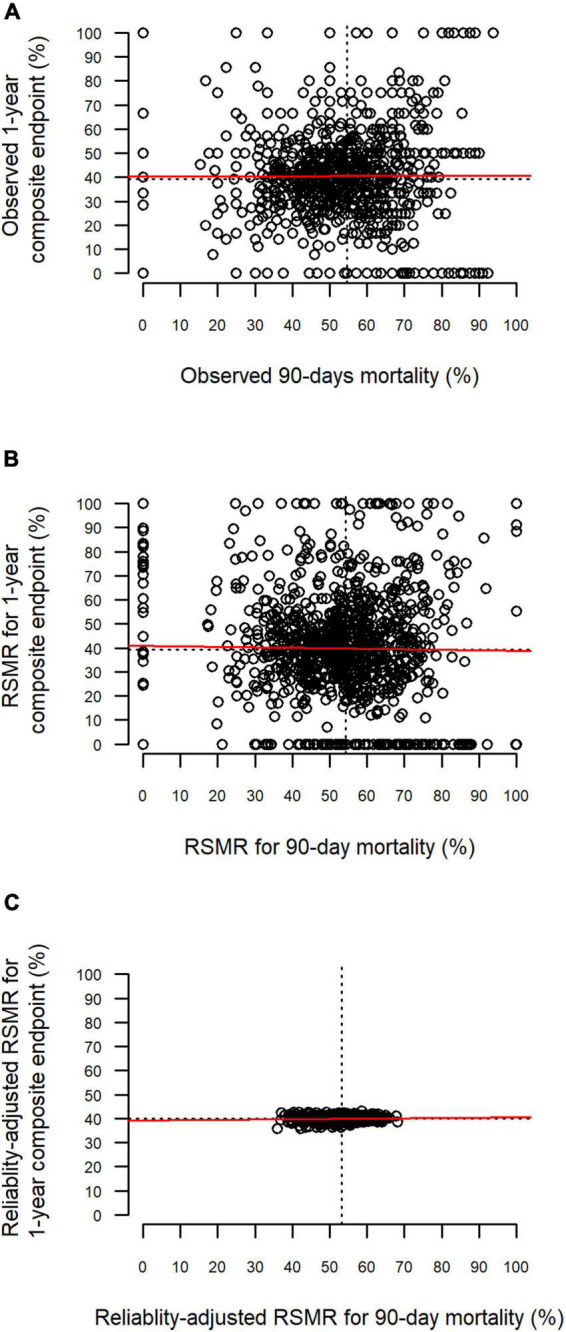
Relationship between 90-day mortality and the 1-year composite endpoint. Each point represents one hospital. The red line represents the linear relationship based on least-squares estimation. One-year composite endpoint defined by death or increased dependency on chronic care during 1 year after discharge. **(A)** Relationship between observed rates. **(B)** Relationship between risk-standardized rates (RSMR). **(C)** Relationship between reliability-adjusted RSMR.

The relationships between the different rates for 90-day mortality and the 1-year composite endpoint are depicted in Figure 3. There were no substantial correlations observed between the two endpoints (observed rates: ρ = 0, *p* = 0.99; RSMR: ρ = 0.017, *p* = 0.56; reliability-adjusted RSMR: ρ = 0.067; *p* = 0.026).

## 4. Discussion

### 4.1. Summary of results

This is the first study on the development of health claims based risk-adjusted quality indicators for acute sepsis care, which incorporate both 90-day mortality as well as long-term outcomes on mortality and functional dependency. The risk-adjustment models relied on pre-existing conditions actually measured before hospitalization and showed a decent predictive validity. There was no evidence for correlation between short-term and long-term outcomes at the level of hospitals.

### 4.2. Interpretation

Regarding 90-day mortality, the predictive validity of our model was comparable to previously reported administrative data based models on short-term mortality after acute treatment of sepsis (10–12). Even risk-adjustment models, which were based on clinical data or a mix of clinical and administrative data, did–with AUCs between 0.75 and 0.78–not achieve relevantly higher discrimination (22, 24, 44). The effects of risk factors in the model are similar to previously reported risk-models for sepsis mortality (10–12, 22). Protective effects of some comorbidities on short-term mortality–namely obesity, depression, and hypertension–have also been shown in these studies as well as in studies on the Elixhauser comorbidity index conducted among representative samples of hospital patients (26, 45, 46). It has been argued that these seemingly protective effects reflect a bias in coding, where relatively healthy patients without severe comorbidities have a higher chance of having these milder comorbidities coded compared to more severely ill patients. We found that also pre-existing long-term mechanical ventilation had a protective effect on risk of 90-day mortality. This might be due to the intense ongoing monitoring of these patients, which might have allowed early detection and adequate treatment of sepsis and thereby prevention of acute deterioration and death (47).

To our knowledge, no other risk-adjustment model on long-term outcomes of sepsis care based on administrative data exists. Moreover, prediction scores for long-term outcomes based on clinical data are also lacking (48, 49). Based on an ICU-registry, Shankar-Hari et al. developed a prognostic score for the composite endpoint of 1-year mortality or re-hospitalization, which showed an AUC of 0.68–comparable to the AUC of 0.675 estimated for our model on 1-year mortality or increased dependency on chronic care. As expected, we found that older patients with comorbid conditions tended to have a higher risk of long-term mortality or dependency on chronic care after having survived 90 days post-discharge. Pre-existing long-term mechanical ventilation increased the risk of long-term mortality or care dependency, likewise because these patients have a reduced survival time in general. The protective effect of pre-existing dependency on chronic care might simply indicate that the risk to develop dependency on chronic care for the first time is higher compared to switching to a higher degree of dependency, if a patient was already receiving chronic care.

Patients with sepsis can suffer from a broad spectrum of clinical sequelae in the areas of physical disability, cognitive impairment, mental health impairment, recurrent infection and sepsis, exacerbation of chronic conditions, all of which decrease overall functioning and quality of life (4, 19). Since concrete sequelae can be highly variable across patients and their measurement in administrative data is dependent on validity of ICD-coding (19), we selected the increase in the dependency on chronic care as an objective indicator of cumulative, overall functioning. It has to be acknowledged that long-term outcomes of acute care cannot solely be attributed to the initially treating hospital, since they are also influenced by other health-care providers responsible for the further treatment as well as other factors (50). Little is known on how to enhance long-term recovery of survivors during acute in-patient care on the ICU or the ward (4, 51, 52). Because of this, attributing long-term outcomes of sepsis care to the initial hospitalization is especially problematic. This might explain why 90-day mortality and the composite 1-year endpoint did not correlate in our study. Therefore, short-term mortality may serve as the better indicator of quality of acute sepsis care. On the other hand, a reduction in short-term mortality after changes in treatment regiments might come at the cost of an increase in long-term mortality or worsening of other patient-centered outcomes (4, 16). Including indicators of long-term sequelae to a measurement of the quality of care can therefore help to interpret differences between providers as well as changes across time both in quality assurance and population surveillance (16).

The problem of reliability adjustment has been extensively discussed in methodological literature (35, 38–42), but is currently not applied in prominent voluntary performance measurement programs in Germany or the methodology of the mandatory quality indicators for German hospitals (20, 53, 54). When case numbers are small, it is hard to tell if extremely high or low outcome rates are due to chance or true differences in quality of care (39). Since there typically is a time-lag between data collection and report of quality indicators, the validity of a quality indicator to predict future performance is important. Shrinkage estimators shrink the estimate of the rate toward the average rate of the population, with the amount of shrinkage negatively proportional to the number of cases. This shrunken estimator has been shown to be a better predictor for future performance compared to classical methods based on logistic regression (38, 39). In our study, reliability adjustment resulted in a great reduction in variability in endpoints between hospitals compared to raw endpoints as well as compared to non-reliability-adjusted RSMR. This effect was stronger for the 1-year-composite endpoint, given the smaller case numbers per hospital. Shrunken estimators are especially relevant for quality indicators used in public reporting or pay-for-performance systems, where wrongfully assigning low-volume hospitals a below-average performance due to unreliable estimates would result in unwarranted negative financial consequences. Nevertheless, if the aim is the identification of possible shortcomings of care for further investigation in continuous quality improvement programs or by peer-reviews, it might be important not to miss possible signals and therefore to also take non-reliability-adjusted estimates into consideration–especially if case-numbers per hospital tend to be low (55).

### 4.3. Practical usage of the presented methodology

The developed methodology could be used for the purposes of quality measurement and between-hospital comparisons. It could also be useful in population surveillance to monitor the changes in mortality and morbidity on a population level across time in Germany (19, 56). It can be applied to data of the same structure and content, i.e., health claims data of the AOK or of other German public health care insurers, which all obtain largely the same information collected for administrative purposes. The German Institute for Quality and Efficiency in Health Care has recently been instructed to develop mandatory quality indicators for acute sepsis care by the Joint Federal Committee–the institution responsible for quality assurance of health care in Germany (57). Since combined data of all public health insurers are used to calculate mandatory quality indicators of German hospitals, the problem of low case volume and lacking reliability of estimates might be overcome in this context. Thus, the presented methodology could be applied both in voluntary performance measurement in the context of the QSR-program as well as in the context of mandatory quality assurance for German hospitals. For any purpose, the risk-adjustment models should be recalculated using the respective current population and reference period to allow adequate comparisons. For this reason, we refrained from validating the reported model in a separate external cohort, since the methodology is not intended to be used this way.

### 4.4. Strengths and limitations

Our study has several strengths. It presents the first claims-based risk-adjusted quality indicators for long-term outcome of acute sepsis care, which include risk factors measured before the index hospitalization. This is an important improvement compared to previous claims-based models, which only relied on information documented during the hospital stay (10–12). Operationalization of variables was done in a rigorous process, based on a multiprofessional panel of experts who care for patients with sepsis (19). Based on the large sample size, complex risk-adjustment models with decent predictive validity were derived.

Our study also has several limitations, mostly associated with general shortcomings of administrative health data (7). These data are limited in content and provide no information on vital signs, microbiological results or medication during the hospital stay. Therefore, several known risk factors for short-term and long-term patient outcomes, like presenting signs and symptoms or severity of initial critical illness could not be assessed (49, 51). On the other hand, predictive validity was comparable to models incorporating clinical data (22, 24, 44, 49), and previous studies found no relevant differences between risk-standardized rates based solely on administrative data compared to administrative data enhanced by clinical information (11, 35). Administrative data are also limited in accuracy and completeness of coding of information (7). This results in possible information biases affecting the case selection, as well as identification of risk factors and outcomes. Lacking accuracy and mostly undercoding has been described for the ICD-coding of sepsis (58, 59). This can have huge effects on the validity of performance measures and provider comparisons, especially if the quality of coding varies across hospitals (7, 60). Based on our data, in average only one third of German patients coded with sepsis per hospital and year were included, which–together with the problem of undercoding of sepsis–results in decreased reliability of estimates and reduced power to detect hospitals, which are outliers in risk-adjusted endpoints. Our study was based on data gathered before the introduction of the new Sepsis-3 definitions (1). Therefore, we included patients with ICD-codes for sepsis with organ dysfunction or septic shock according to sepsis-1/sepsis-2 definitions, since severe sepsis according to old sepsis definitions shows a high overlap with the new definitions (61). We used a composite outcome of mortality and increase in long-term morbidity. While this allows to address the competing risk between both endpoints (21), it leads to difficulties in interpreting differences in hospital performance. Multistate models provide a comprehensive methodology to address competing risks between outcomes (62). But since there does not yet exist a method to obtain hospital-specific estimates of risk-adjusted quality indicators from such models, further methodological development is needed to apply them in the context of provider comparisons.

## 5. Conclusion

We presented a methodology for claims-based risk-adjusted quality indicators on short-term and long-term mortality and morbidity after acute sepsis care. Beside the limitations of administrative health data, this methodology could provide a valuable tool in assessing and monitoring outcome quality achieved by German hospitals caring for patients with sepsis. Future studies should recalculate the risk-adjustment models based on current data incorporating the new Sepsis-3 definitions and may embrace multi state modeling to address mortality and morbidity jointly. To increase reliability and validity of measures of outcome quality, data sources should cover the full population and hospitals need to improve ICD-10-coding of sepsis.

## Data availability statement

The data analyzed in this study is subject to the following licenses/restrictions: The data used in this study cannot be made available in the manuscript, the Supplementary material, or in a public repository due to German data protection laws (Bundesdatenschutzgesetz). Therefore, they are stored on a secure drive in the Wissenschaftliches Institut der AOK to facilitate replication of the results. Generally, access to data of statutory health insurance funds for research purposes is possible only under the conditions defined in the German Social Law (SGB V §287). Requests for data access can be sent as a formal proposal, specifying the recipient and purpose of the data transfer, to the appropriate data protection agency. Access to the data used in this study can only be provided to external parties under the conditions of the cooperation contract of this research project and after written approval by the AOK. For assistance in obtaining access to the data, please contact wido@wido.bv.aok.de.

## Ethics statement

The studies involving human participants were reviewed and approved by Ethik-Kommission der Friedrich-Schiller-Universität Jena. Written informed consent for participation was not required for this study in accordance with the national legislation and the institutional requirements.

## Author contributions

DS, CF-S, and MS: concept and design. CF-S, NR, MS, CG, and LW: acquisition of data. LW, DS, NR, and PS: statistical analysis and planning. LW and DS: drafting of the manuscript. DS: supervision. CG, AS, and PS: administrative support. LW: conduction and had full access to all of the data in the study and takes responsibility for the integrity of the data and the accuracy of the data analysis. All authors contributed to interpretation of results and critical revision of the manuscript for important intellectual content. All authors contributed to the article and approved the submitted version.
