# Supplementary material for: Development and validation of risk-adjusted quality indicators for the long-term outcome of acute sepsis care in German hospitals based on health claims data
Source: Front Med (Lausanne). 2023 Jan 9;9:1069042. doi: 10.3389/fmed.2022.1069042 (PMC9868402; doi:10.3389/fmed.2022.1069042)
Supplement: Supplementary file 2 [file Table_1.DOCX]

Supplementary Material 1: Definition of variables

# Case identification: Definitions and Codes

# During the complete observation period, sepsis coding was based on the sepsis-1/2 criteria in Germany. Patients with an ICD-10-GM code for severe sepsis or septic shock were included in this secondary analysis.

| Severe sepsis |  |
| --- | --- |
| ICD-10-GM Codes: |  |
| R65.1 | Systemic Inflammatory Response Syndrome of infectious origin with organ failure |

| Septic shock |  |
| --- | --- |
| ICD-10-GM Codes: |  |
| R57.2 | Septic shock |

# Outcomes

| 90-days mortality | short-term endpoint 90-days mortality after hospital discharge |
| --- | --- |
|  |  |
| 1-year composite endpoint | long-term composite (binary) outcome: 1-year mortality OR increase in the dependency on chronic care during the year after hospital discharge from index hospitalization  increase need of chronic care:   - increase of nursing care level - new transition to a long-term nursing home |

# Risk-factors

## Demographics

| Age | Age of the patient at the time of discharge date index stay |
| --- | --- |
| Gender | Gender of the patient at the time of discharge date index stay |

## Comorbidities

Comorbidities defined according to Charlson and Elixhauser Comorbidity Index with reference to Quan et. al (1) in the year prior to index hospitalizations.

Charlson Comorbidity Index for

| Cerebrovascular disease |
| --- |
| Dementia |
| Mild liver disease |
| Moderate or severe liver disease |
| Myocardial infarction |
| Peptid ulcer disease |
| Rheumatic disease |

Elixhauser Comorbidity Index for

| Alcohol abuse |
| --- |
| Blood loss anemia |
| Cardiac arrhythmias |
| Chronic pulmonary disease |
| Coagulopathy |
| Congestive heart failure |
| Deficiency anemia |
| Depression |
| Diabetes, complicated |
| Diabetes, uncomplicated |
| Drug abuse |
| Fluid and electrolyte disorders |
| Hypertension, complicated |
| Hypertension, uncomplicated |
| Hypothyroidism |
| Leucaemia |
| Lymphoma |
| Metastatic cancer |
| Obesity |
| Other neurological disorders |
| Paralysis |
| Peripheral vascular disorders |
| Psychoses |
| Pulmonary circulation disorders |
| Renal failure |
| Solid tumor without metastasis |
| Valvular disease |
| Weight loss |

## Pre-existing conditions and treatments

| Pre-existing immobility in the year prior to index hospitalization |  |
| --- | --- |
| ICD-10-GM Codes: |  |
| R26.2 | Difficulty in walking, not elsewhere classified |
| R26.3 | Immobility |
| R29.6 | Tendency to fall, not elsewhere classified |
| Z99.3 | Dependence on wheelchair |
| Z74.0 | Need for assistance due to reduced mobility |

| Pre-existing dependency on chronic care |  |
| --- | --- |
|  | Nursing home in the year prior to index hospitalization |
|  | Care Level > 0 in the year prior to index hospitalization |

| Pre-existing long-term mechanical ventilation in the year prior to index hospitalization |  |
| --- | --- |
| ICD-10-GM Codes: |  |
| Z99.0 | Dependence on aspirator |
| Z99.1 | Dependence on respirator |
| OPS Codes: |  |
| 8-713 | Mechanical ventilation and respiratory support in adults |
| 8-712 | Mechanical ventilation and respiratory support in children and adolescents |
| 8-714 | Special procedure for mechanical ventilation in the case of severe respiratory failure |
| 8-70 | Access for mechanical ventilation and measures to maintain the airway |
| 8-71 | Mechanical ventilation and respiratory support via a mask or tube and ventilation weaning |

| Pre-existing renal replacement therapy in the year prior to index hospitalization |  |
| --- | --- |
| ICD-10-GM Codes: |  |
| Z99.2 | Dependence on renal dialysis |
| Z49 | Care involving dialysis |
| OPS Codes: |  |
| 5-392 | Creation of an arteriovenous fistula |
| 8-853 | Haemofiltration |
| 8-854 | Haemodialysis |
| 8-855 | Haemodiafiltration |
| 8-857 | Peritoneal dialysis |
| Statutory scale of fees for physicians (GOÄ) Codes: |  |
| 13602 | Flat rate supplementary fee for continuous care of a patient requiring dialysis |
| 13610 | Flat rate supplementary fee for medical care in the case of haemodialysis, peritoneal dialysis and special procedures |
| 13611 | Flat rate supplementary fee for medical care in the case of peritoneal dialysis |
| 4562 | Flat rate supplementary fee for continuous care of a patient requiring dialysis |
| 4564 | Flat rate supplementary fee for paediatric nephrology care when carrying out haemodialysis |
| 4565 | Flat rate supplementary fee for paediatric nephrology care when carrying out peritoneal dialysis |
| 40815 | Flat rate fee for dialysis in patients up to the age of 18 years at their place of residence |
| 40816 | Flat rate fee for peritoneal dialysis in patients up to the age of 18 years |
| 40817 | Flat rate fee for peritoneal dialysis in patients up to the age of 18 years at their place of residence |
| 40818 | Flat rate fee for haemodialysis in patients up to the age of 18 years during a holiday or other absence |
| 40819 | Flat rate fee for peritoneal dialysis in patients up to the age of 18 years during a holiday or other absence |
| 40823 | Flat rate fee for dialysis in insured persons from the age of 18 years |
| 40824 | Flat rate fee for dialysis in insured persons from the age of 18 years at their place of residence |
| 40825 | Flat rate fee for peritoneal dialysis in insured persons from the age of 18 years |
| 40826 | Flat rate fee for peritoneal dialysis in insured persons from the age of 18 years at their place of residence |
| 40827 | Flat rate fee for intermittent peritoneal dialysis in insured persons from the age of 18 years at their place of residence |
| 40828 | Flat rate fee for dialysis from the age of 18 years during a holiday or work-related stay |
| 40829 | Supplement to flat rate fee 40823 or 40825 for insured persons aged 59-69 years |
| 40830 | Supplement to flat rate fee 40824, 40826 and 40827 for insured persons aged 59-69 years |
| 40831 | Supplement to flat rate fee 40823 or 40825 for insured persons aged 69-79 years |
| 40832 | Supplement to flat rate fee 40824, 40826 and 40827 for insured persons aged 69-79 years |
| 40833 | Supplement to flat rate fee 40823 or 40825 for insured persons from 79 years of age |
| 40834 | Supplement to flat rate fee 40824, 40826 and 40827 for insured persons from 79 years of age |
| 40835 | Supplement to flat rate fee 40816, 40823 or 40825 for dialysis in a patient with an infection |
| 40836 | Supplement to flat rate fee 40815, 40817, 40818, 40819, 40824, 40826 to 40828 for dialysis in a patient with an infection |
| 40837 | Supplement to flat rate fee 40816 or 40825 for intermittent peritoneal dialysis |
| 40838 | Supplement to flat rate fee 40817, 40819, 40827 or 40828 for intermittent peritoneal dialysis |

| Prior palliative treatment in the year prior to index hospitalization |  |
| --- | --- |
| ICD-10-GM Codes: |  |
| Z51.5 | Palliative care |
| OPS: |  |
| 8-982 | Palliative medical complex treatment |
| 8-98e | Specialized inpatient palliative medical complex treatment |
| 8-98h | Specialized palliative medical complex treatment through a palliative care service |
| Statutory scale of fees for physicians (GOÄ) Codes: |  |
| 1425 | Initial care in specialized outpatient palliative care |
| 1426 | Follow-up prescription for continuation of the specialized outpatient palliative care |
| 3370 | Palliative medical initial diagnosis |
| 3371 | Supplementary fee for palliative medical care in the medical practice |
| 3372 | Supplementary fee for palliative medical care in the home |
| 3373 | Supplementary fee for palliative medical care in the home |
| 1425 | Initial prescription for specialized outpatient palliative care |
| 1426 | Follow-up prescription for continuation of the specialized outpatient palliative care |
| 3370 | Palliative medical initial diagnosis of patient status including treatment plan |
| 3371 | Supplementary fee to the insured persons flat rate 03000 for palliative medical care of the patient in the medical practice |
| 3372 | Supplementary fee to Catalogue of Tariffs for Physicians code 01410 or 01413 for palliative medical care in the home |
| 3373 | Supplementary fee to Catalogue of Tariffs for Physicians code 01411, 01412 or 01415 for palliative medical care in the home |
| 4370 | Palliative medical initial diagnosis |
| 4371 | Supplementary fee to the insured persons flat rate 04000 for palliative medical care of the patient in the medical practice |
| 4372 | Supplementary fee to Catalogue of Tariffs for Physicians code 01410 or 01413 for palliative medical care in the home |
| 4373 | Supplementary fee to Catalogue of Tariffs for Physicians code 01411, 01412 or 01415 for palliative medical care in the home |
| 37302 | Supplementary fee to the insured persons flat rate or basic flat rate for the coordinating panel doctor |
| 37314 | Consultation discussion doctor with an additional designation palliative medicine |
| 37318 | Telephone consultation |
| 37300 | Palliative medical initial diagnosis of patient status including treatment plan |
| 37305 | Supplementary fee to tariff codes 01410 and 01413 for palliative medical care in the home |
| 37306 | Supplementary fee to tariff codes 01411 01412 and 01415 for palliative medical care in the home |
| 37317 | Supplementary fee to tariff code 37302 for accessibility and willingness to visit in critical phases |
| 37320 | Case conference |

| Hospital length of stay in the year prior to index hospitalization (days) |  |
| --- | --- |
|  | 0 day |
|  | 1 day |
|  | >1 day and <6 days |
|  | ≥ 6 days and < 10 days |
|  | >10 days |

## Clinical characteristics of the infection

Assessed at discharge from the index treatment

| Sepsis as primary diagnosis |  |
| --- | --- |
| ICD-10-GM Codes: |  |
| A02.1 | Salmonella sepsis |
| A20.0 | Bubonic plague |
| A20.7 | Septicaemic plague |
| A21.7 | Generalized tularaemia |
| A22.7 | Anthrax sepsis |
| A24.1 | Acute or fulminating melioidosis |
| A26.7 | Erysipelothrix sepsis |
| A28.2 | Extraintestinal yersiniosis |
| A32.7 | Listerial sepsis |
| A39.1 | Waterhouse-Friderichsen syndrome |
| A39.2 | Acute meningococcal sepsis |
| A39.3 | Chronic meningogoccal sepsis |
| A39.4 | Meningococcaemia, unspecified |
| A40 | Streptococcal sepsis |
| A41 | Other sepsis |
| A42.7 | Actinomycotic sepsis |
| A48.3 | Toxic shock syndrome |
| A49.9 | Bacterial infection, unspecified |
| A54.8 | Other gonococcal infections |
| B00.7 | Disseminated herpesviral disease |
| B37.6 | Candidal endocarditis |
| B37.7 | Candidal sepsis |
| B49 | Unspecified mycosis |
| O75.3 | Other infection during labour |
| O85 | other puerperal infections |
| R65.0 | Systemic Inflammatory Response Syndrome of infectious origin without organ failure |
| R57.2 | Septic shock |

| **Focus of infection**  Abdominal infections |  |
| --- | --- |
| ICD-10-GM Codes: |  |
| A00 | Cholera |
| A01 | Typhoid and paratyphoid fevers |
| A02 | Other salmonella infections |
| A03 | Shigellosis |
| A04 | Other bacterial intestinal infections |
| A05 | Other bacterial foodborne intoxications, not elsewhere classified |
| A06 | Amoebiasis |
| A07 | Other protozoal intestinal diseases |
| A08 | Viral and other specified intestinal infections |
| A09 | Other gastroenteritis and colitis of infectious and unspecified origin |
| K35 | Acute appendicitis |
| K37 | Unspecified appendicitis |
| K36 | Other appendicitis |
| K57.02 | Diverticular disease of small intestine with perforation and abscess without bleeding |
| K57.03 | Diverticular disease of small intestine with perforation and abscess with bleeding |
| K57.12 | Diverticular disease of small intestine without perforation or abscess without bleeding |
| K57.13 | Diverticular disease of small intestine without perforation or abscess wit bleeding |
| K57.22 | Diverticular disease of large intestine with perforation and abscess without bleeding |
| K57.23 | Diverticular disease of large intestine with perforation, abscess and bleeding |
| K57.32 | Diverticular disease of large intestine without perforation or abscess wihout bleeding |
| K57.33 | Diverticular disease of large intestine without perforation or abscess wit bleeding |
| K57.42 | Diverticular disease of both small and large intestine with perforation and abscess without bleeding |
| K57.43 | Diverticular disease of both small and large intestine with perforation, abscess and bleeding |
| K57.52 | Diverticular disease of both small and large intestine without perforation or abscess or bleeding |
| K57.53 | Diverticular disease of both small and large intestine without perforation or abscess with bleeding |
| K57.82 | Diverticular disease of intestine, part unspecified, with perforation and abscess without bleeding |
| K57.83 | Diverticular disease of intestine, part unspecified with perforation, abscess and bleeding |
| K57.92 | Diverticular disease of intestine, part unspecified, without perforation, abscess or bleeding |
| K57.93 | Diverticular disease of intestine, part unspecified, without perforation or abscess with bleeding |
| K61 | Abscess of anal and rectal regions |
| K65 | Peritonitis |
| K67 | Disorders of peritoneum in infectious diseases classified elsewhere |
| K63.0 | Abscess of intestine |
| K63.1 | Perforation of intestine (nontraumatic) |
| K75.0 | Abscess of liver |
| K75.1 | Phlebitis of portal vein |
| K81.0 | Cholecystitis |
| K77.0 | Liver disorders in infectious and parasitic diseases classified elsewhere |
| U69.40! | Recurrent infection due to Clostridium difficile |

| **Focus of infection** |  |
| --- | --- |
| Respiratory tract |  |
| ICD-10-GM Codes: |  |
| J01 | Acute sinusitis |
| J02 | Acute pharyngitis |
| J03 | Acute tonsillitis |
| J04 | Acute laryngitis and tracheitis |
| J06 | Acute upper respiratory infections of multiple and unspecified sites |
| J05 | Acute obstructive laryngitis [croup] and epiglottitis |
| J09 | Influenza due to identified zoonotic or pandemic influenza virus |
| J10 | Influenza due to identified seasonal influenza virus |
| J11 | Influenza, virus not identified |
| J12 | Viral pneumonia, not elsewhere classified |
| J13 | Pneumonia due to Streptococcus pneumoniae |
| J14 | Pneumonia due to Haemophilus influenzae |
| J15 | Bacterial pneumonia, not elsewhere classified |
| J16 | Pneumonia due to other infectious organisms, not elsewhere classified |
| J17 | Pneumonia in diseases classified elsewhere |
| J18 | Pneumonia, organism unspecified |
| J20 | Acute bronchitis |
| J21 | Acute bronchiolitis |
| J22 | Unspecified acute lower respiratory infection |
| J44.0 | Chronic obstructive pulmonary disease with acute lower respiratory infection |
| J44.1 | Chronic obstructive pulmonary disease with acute exacerbation, unspecified |
| J86 | Pyothorax |
| J85 | Abscess of lung and mediastinum |
| A15 | Respiratory tuberculosis, bacteriologically or histologically confirmed |
| A16 | Respiratory tuberculosis, not confirmed bacteriologically or histologically |
| U69.00 | Hospital-acquired pneumonia in other diseases classified elsewhere |
| A36 | Diphtheria |
| A37 | Whooping cough |
| B38 | Coccidioidomycosis |
| B39 | Histoplasmosis |

| **Focus of infection**  Central nervous system infection |  |
| --- | --- |
| ICD-10-GM Codes: |  |
| A39 | Meningococcal infection |
| G00 | Bacterial meningitis, not elsewhere classified |
| G01 | Meningitis in bacterial diseases classified elsewhere |
| G02 | Meningitis in other infectious and parasitic diseases classified elsewhere |
| G03 | Meningitis due to other and unspecified causes |
| G04 | Encephalitis, myelitis and encephalomyelitis |
| G05* | Encephalitis, myelitis and encephalomyelitis in diseases classified elsewhere |
| G06 | Intracranial and intraspinal abscess and granuloma |
| G07* | Intracranial and intraspinal abscess and granuloma in diseases classified elsewhere |
| G08 | Intracranial and intraspinal phlebitis and thrombophlebitis |
| A17+ | Tuberculosis of nervous system |
| A81 | Atypical virus infections of central nervous system |
| A83 | Mosquito-borne viral encephalitis |
| A84 | Tick-borne viral encephalitis |
| A85 | Other viral encephalitis, not elsewhere classified |
| A86 | Unspecified viral encephalitis |
| A87 | Viral meningitis |
| A88 | Other viral infections of central nervous system, not elsewhere classified |
| A89 | Unspecified viral infection of central nervous system |

| **Focus of infection**  Cardiovascular system infection |  |
| --- | --- |
| ICD-10-GM Codes: |  |
| I32 | Pericarditis in diseases classified elsewhere |
| I33 | Acute and subacute endocarditis |
| I39 | Endocarditis and heart valve disorders in diseases classified elsewhere |
| I40 | Acute myocarditis |
| I41 | Myocarditis in diseases classified elsewhere |
| I80 | Thombosis, phlebitis and thrombophlebitis |
| I38 | Endocarditis, valve unspecified |
| I98.1 | Cardiovascular disorders in other infectious and parasitic diseases classified elsewhere |

| **Focus of infection**  Device-related infections |  |
| --- | --- |
| ICD-10-GM Codes: |  |
| T82.6 | Infection and inflammatory reaction due to cardiac valve prosthesis |
| T82.7 | Infection and inflammatory reaction due to other cardiac and vascular devices, implants and grafts |
| T83.5 | Infection and inflammatory reaction due to prosthetic device, implant and graft in urinary system |
| T83.6 | Infection and inflammatory reaction due to prosthetic device, implant and graft in genital tract |
| T84.5 | Infection and inflammatory reaction due to internal joint prosthesis |
| T84.6 | Infection and inflammatory reaction due to internal fixation device [any site] |
| T84.7 | Infection and inflammatory reaction due to other internal orthopaedic prosthetic devices, implants and grafts |
| T85.7 | Infection and inflammatory reaction due to other internal prosthetic devices, implants and grafts |

| **Focus of infection**  Other or unspecified |  |
| --- | --- |
| ICD-10-GM Codes: |  |

| A18 | Tuberculosis of other organs |
| --- | --- |
| A19 | Miliary tuberculosis |
| A20 | Plague |
| A21 | Tularaemia |
| A22 | Anthrax |
| A23 | Brucellosis |
| A24 | Glanders and melioidosis |
| A25 | Rat-bite fevers |
| A26 | Erysipeloid |
| A27 | Leptospirosis |
| A28 | Other zoonotic bacterial diseases, not elsewhere classified |
| A32 | Listeriosis |
| A38 | Scarlet fever |
| A42 | Actinomycosis |
| A43 | Nocardiosis |
| A44 | Bartonellosis |
| A48 | Other bacterial diseases, not elsewhere classified |
| A49 | Bacterial infection of unspecified site |
| A50 | Congenital syphilis |
| A54 | Gonococcal infection |
| A65 | Nonvenereal syphilis |
| A69.0 | Necrotizing ulcerative stomatitis |
| A69.1 | Other Vincent infections |
| A69.2 | Lyme disease |
| A69.8 | Other specified spirochaetal infections |
| A69.9 | Spirochaetal infection, unspecified |
| A74 | Other diseases caused by chlamydiae |
| A75 | Typhus fever |
| A77 | Spotted fever [tick-borne rickettsioses] |
| A78 | Q fever |
| A79 | Other rickettsioses |
| A80 | Acute poliomyelitis |
| A90 | Dengue fever [classical dengue] |
| A91 | Dengue haemorrhagic fever |
| A92 | Other mosquito-borne viral fevers |
| A93 | Other arthropod-borne viral fevers, not elsewhere classified |
| A94 | Unspecified arthropod-borne viral fever |
| A95 | Yellow fever |
| A96 | Arenaviral haemorrhagic fever |
| A97 | Dengue |
| A98 | Other viral haemorrhagic fevers, not elsewhere classified |
| A99 | Unspecified viral haemorrhagic fever |
| B01 | Varicella [chickenpox] |
| B02 | Zoster [herpes zoster] |
| B03 | Smallpox |
| B04 | Monkeypox |
| B05 | Measles |
| B06 | Rubella |
| B25 | Cytomegaloviral disease |
| B26 | Mumps |
| B27 | Infectious mononucleosis |
| B33 | Other viral diseases, not elsewhere classified |
| B34 | Viral infection of unspecified site |
| B37 | Candidiasis |
| B40 | Blastomycosis |
| B41 | Paracoccidioidomycosis |
| B42 | Sporotrichosis |
| B43 | Chromomycosis and chromomycotic abscess |
| B44 | Aspergillosis |
| B45 | Cryptococcosis |
| B46 | Zygomycosis |
| B48 | Other mycoses, not elsewhere classified |
| B49 | Unspecified mycosis |
| B50 | Plasmodium falciparum malaria |
| B51 | Plasmodium vivax malaria |
| B52 | Plasmodium malariae malaria |
| B53 | Other parasitologically confirmed malaria |
| B54 | Unspecified malaria |
| B55 | Leishmaniasis |
| B58 | Toxoplasmosis |
| B60 | Other protozoal diseases, not elsewhere classified |
| B64 | Unspecified protozoal disease |
| B67 | Echinococcosis |
| B95 | Streptococcus and staphylococcus as the cause of diseases classified to other chapters |
| B96 | Other specified bacterial agents as the cause of diseases classified to other chapters |
| B97 | Viral agents as the cause of diseases classified to other chapters |
| B98 | Other specified infectious agents as the cause of diseases classified to other chapters |
| B99 | Other and unspecified infectious diseases |
| M00 | Pyogenic arthritis |
| M01 | Direct infections of joint in infectious and parasitic diseases classified elsewhere |
| M86 | Osteomyelitis |
| T80.2 | Infections following infusion, transfusion and therapeutic injection |
| T81.4 | Infection following a procedure, not elsewhere classified |
| T88.0 | Infection following immunization |

| **Focus of infection**  Genitourinary system infection |  |
| --- | --- |
| ICD-10-GM Codes: |  |
| N10 | Acute tubulo-interstitial nephritis |
| N15.1 | Renal and perinephric abscess |
| N15.9 | Renal tubulo-interstitial disease, unspecified |
| N34 | Urethritis and urethral syndrome |
| N30 | Cystitis |
| N39.0 | Urinary tract infection, site not specified |
| N41 | Inflammatory diseases of prostate |
| N45 | Orchitis and epididymitis |
| N48.2 | Other inflammatory disorders of penis |
| N49 | Inflammatory disorders of male genital organs, not elsewhere classified |
| N70 | Salpingitis and oophoritis |
| N71 | Inflammatory disease of uterus, except cervix |
| N72 | Inflammatory disease of cervix uteri |
| N73 | Other female pelvic inflammatory diseases |
| N74 | Female pelvic inflammatory disorders in diseases classified elsewhere |
| N75 | Diseases of Bartholin gland |
| N76 | Other inflammation of vagina and vulva |
| N77 | Vulvovaginal ulceration and inflammation in diseases classified elsewhere |
| N61 | Inflammatory disorders of breast |
| N98.0 | Infection associated with artificial insemination |
| A59 | Trichomoniasis |
| A55 | Chlamydial lymphogranuloma (venereum) |
| A56 | Other sexually transmitted chlamydial diseases |

| **Focus of infection**  Wound/soft tissue infection |  |
| --- | --- |
| ICD-10-GM Codes: |  |
| A46 | Erysipelas |
| B47 | Mycetoma |
| L03 | Phlegmon |
| L04 | Acute lymphadenitis |
| L08 | Other local infections of skin and subcutaneous tissue |
| L05 | Pilonidal cyst |
| B00 | Herpesviral [herpes simplex] infections |
| B07 | Viral warts |
| B08 | Other viral infections characterized by skin and mucous membrane lesions, not elsewhere classified |
| B09 | Unspecified viral infection characterized by skin and mucous membrane lesions |
| H05.0 | Acute inflammation of orbit |
| H60.2 | Malignant otitis externa |
| H70.0 | Acute mastoiditis |
| J36 | Peritonsillar abscess |
| J39.0 | Retropharyngeal and parapharyngeal abscess |
| J39.1 | Other abscess of pharynx |
| L02 | Cutaneous abscess, furuncle and carbuncle |

## Hospital admission type for the index hospitalization

| Reason for admission |  |
| --- | --- |
|  | emergency admission |
|  | referral by physician or other |
|  | transfer from another hospital |

## Specific treatments during the index hospitalization

| Chemotherapy during index hospitalization  OPS Codes: |  |
| --- | --- |
| 8-54 | Cytostatic chemotherapy, immunotherapy and antiretroviral therapy |

| Complex treatment stroke during index hospitalization  OPS Codes: |  |
| --- | --- |
| 8-981 | Neurological complex treatment of acute stroke |
| 8-98b | Other neurological complex treatment of acute stroke |

| Multidrug-resistant infections |  |
| --- | --- |
| ICD-10-GM Codes: |  |
| U80.! | Grampositive bacteria with specified antibiotic resistance, requiring special therapeutic or hygienic measures |
| U81.! | Gram negative bacteria with specified antibiotic resistanc, requiring special therapeutic or hygienic measures |
| U82.! | Mycobacteria with resistance against TB drugs (first line) |
| U83.! | Candida with resistance against Fluconazole and Voriconazole |
| U84.! | Herpes virus with restistance against antivirals |
| U85! | Human Immunodeficiency Virus with resistance against antivirals or proteinase â€“ inhibitors |
| OPS Codes: |  |
| 8-987 | Complex treatment in the case of colonisation or infection with multidrug-resistant pathogens [MDR] |

References

1. Quan HD, Sundararajan V, Halfon P, Fong A, Burnand B, Luthi JC, et al. Coding algorithms for defining comorbidities in ICD-9-CM and ICD-10 administrative data. Medical Care. 2005;43(11):1130-9.
