# Supplementary material for: Development and validation of risk-adjusted quality indicators for the long-term outcome of acute sepsis care in German hospitals based on health claims data
Source: Front Med (Lausanne). 2023 Jan 9;9:1069042. doi: 10.3389/fmed.2022.1069042 (PMC9868402; doi:10.3389/fmed.2022.1069042)
Supplement: Supplementary file 3 [file Table_2.DOCX]

Supplementary Material 2: Internal validation

**Supplemental Table 1. Uncorrected and corrected estimates of the validity measures for 90-days mortality.**

| Validity measures | Uncorrected estimates | Corrected estimates using bootstrap with subtraction of the mean optimism | Corrected estimates using bootstrap with prediction on out-of-bag samples |
| --- | --- | --- | --- |
| AUC | 0.756 | 0.748 | 0.756 |
| R^2^ | 0.259 | 0.242 | 0.258 |
| Brier Score | 0.200 | 0.203 | 0.200 |
| Calibration Slope | 1.082 | 1.094 | 1.083 |

First column: uncorrected estimates for the validity measures AUC, R^2^, Brier Score and calibration slope. Second column: corrected estimates after the internal validation using a bootstrap approach following advice by Harrell et al. (1). Last column: corrected estimates after internal validation using a bootstrap approach following advice by Kuhn & Johnson (2).

**Supplemental Table 2. Uncorrected and corrected estimates of the validity measures for 1-year composite endpoint.**

| Validity measures | Uncorrected estimates | Corrected estimates using bootstrap with subtraction of the mean optimism | Corrected estimates using bootstrap with prediction on out-of-bag samples |
| --- | --- | --- | --- |
| AUC | 0.702 | 0.675 | 0.702 |
| R^2^ | 0.155 | 0.111 | 0.157 |
| Brier Score | 0.212 | 0.220 | 0.211 |
| Calibration Slope | 1.047 | 0.978 | 1.047 |

First column: uncorrected estimates for the validity measures AUC, R^2^, Brier Score and calibration slope. Second column: corrected estimates after the internal validation using a bootstrap approach following advice by Harrell et al. (1). Last column: corrected estimates after internal validation using a bootstrap approach following advice by Kuhn & Johnson (2).

References:

1. Harrell FE, Jr., Lee KL, Mark DB. Multivariable prognostic models: issues in developing models, evaluating assumptions and adequacy, and measuring and reducing errors. Stat Med. 1996;15(4):361-87.

2. Kuhn M, Johnson K. Applied predictive modeling. New York: Springer; 2013.
